# Loss of Phosphatidylinositol 3-Kinase Activity in Regulatory T Cells Leads to Neuronal Inflammation

**DOI:** 10.4049/jimmunol.2000043

**Published:** 2020-05-15

**Authors:** Anne-Katrien Stark, Elizabeth C. M. Davenport, Daniel T. Patton, Cheryl L. Scudamore, Bart Vanhaesebroeck, Marc Veldhoen, Oliver A. Garden, Klaus Okkenhaug

**Affiliations:** * Laboratory of Lymphocyte Signalling and Development, Babraham Research Campus, Cambridge CB22 3AT, United Kingdom;; † Department of Pathology, University of Cambridge, Cambridge CB2 1QP, United Kingdom;; ‡ Royal Veterinary College, London NW1 0TU, United Kingdom;; § Exepathology, Exmouth EX8 5LQ, United Kingdom;; ¶ UCL Cancer Institute, University College London, London WC1E 6AG, United Kingdom;; ‖ Instituto de Medicina Molecular, Joâo Lobo Antunes, Faculdade de Medicina da Universidade de Lisboa, 1649-028 Lisbon, Portugal; and; # Department of Clinical Sciences and Advanced Medicine, School of Veterinary Medicine, University of Pennsylvania, Philadelphia, PA 19104

## Abstract

Class I PI3K enzymes are critical for the maintenance of effective immunity. In T cells, PI3Kα and PI3Kδ are activated by the TCR and costimulatory receptors, whereas PI3Kγ is activated by G protein–coupled chemokine receptors. PI3Kδ is a key regulator of regulatory T (Treg) cell function. PI3K isoform-selective inhibitors are in development for the treatment of diseases associated with immune dysregulation, including chronic inflammatory conditions, cancer, and autoimmune diseases. Idelalisib (PI3Kδ), alpelisib (PI3Kα), duvelisib (PI3Kδ/γ), and copanlisib (pan-PI3K) have recently been approved for use in cancer treatment. Although effective, these therapies often have severe side effects associated with immune dysregulation and, in particular, loss of Treg cells. Therefore, it is important to gain a better understanding of the relative contribution of different PI3K isoforms under homeostatic and inflammatory conditions. Experimental autoimmune encephalitis is a mouse model of T cell–driven CNS inflammation, in which Treg cells play a key protective role. In this study, we show that PI3Kδ is required to maintain normal Treg cell development and phenotype under homeostatic conditions but that loss of PI3Kδ alone in Treg cells does not lead to autoimmunity. However, combined loss of PI3Kα and PI3Kδ signaling resulted in increased experimental autoimmune encephalitis disease severity. Moreover, mice lacking PI3Kα and PI3Kδ in Treg cells developed spontaneous peripheral nerve inflammation. These results show a key role for PI3K signaling in Treg cell–mediated protection against CNS inflammation.

## Key Points

- PI3Kδ is required for the maintenance of normal Treg cell development and function.
- PI3Kα contributes to maintaining Treg cell function in context of neuroinflammation.
- Loss of PI3Kα and PI3Kδ in Treg cells leads to spontaneous peripheral neuropathy.

## Introduction

Class I PI3Ks convert the membrane phosphoinositide lipid PI(4,5)P_2_ to PI(3,4,5)P_3_ by phosphorylating the 3-OH position on its inositol ring. This leads to the recruitment of PH domain–containing proteins such as AKT to the plasma membrane, resulting in multiple downstream effector pathways, including phosphorylation and nuclear exclusion of Foxo1 and Foxo3a transcription factors and mTORC1/2 activation, which regulate cell survival, proliferation, and migration. The class IA PI3Ks are heterodimers that consist of one of three catalytic subunits: p110α, p110β, and p110δ, each of which associates with a regulatory subunit (p85α, p50α, p55α, p85β, or p55γ). The class IB PI3K consists of the p110γ catalytic subunit, which associates with the p101 or p84 regulatory subunit. The functional enzyme heterodimers are referred to as PI3Kα, PI3Kβ, PI3Kδ, or PI3Kγ, according to the catalytic subunit. The PI3K catalytic subunit isoforms differ in their tissue distribution and function; whereas p110α and p110β are ubiquitously expressed, p110δ and p110γ expression is enriched in immune cells. In general, class IA PI3Ks are activated downstream of tyrosine kinase–coupled receptors, whereas PI3Kγ is activated by G protein–coupled receptors, although exceptions have been identified such as the activation of PI3Kβ downstream of G protein–coupled receptors (1–3). PI3K-mediated signaling is tightly controlled by phosphatases; Pten dephosphorylates PI(3,4,5)P_3_ at the 3-OH position to maintain homeostatic PI(4,5)P_2_ levels, whereas SHIP phosphatases dephosphorylate the 5-OH position to yield PI(3,4)P_2._. In addition, PHLPP phosphatases dephosphorylate pAkt, providing a further level of control downstream of PI3K activation. The class IA PI3Ks play differential roles in the regulation of immune responses. Although p110β plays an important role in myeloid cell development and function, its expression level is low in lymphocytes (2, 4, 5). The main class IA PI3K isoforms expressed in T cells are p110δ followed by p110α, whereas p110β is barely detectable (1, 6, 7).

Normal class I PI3K signaling through the p110δ isoform is essential for effective B and T cell–mediated immunity; both PI3Kδ inhibition and hyperactivation result in defective adaptive immune responses (8). In T cells, p110δ is the main isoform activated downstream of the TCR and is required for TCR and IL-2 signaling as well as costimulation and promotes the differentiation and function of the CD4^+^ Th1, Th2, and Th17 cell subsets (1, 9–12). However, the role of PI3Kδ signaling in regulatory T (Treg) cell development and function is more complex and not completely understood (13, 14). Treg cells develop in the thymus in response to intermediate self-antigen avidity (thymic Treg cells). In addition, Treg cells can develop in the periphery from naive T cells (peripheral Treg [pTreg] cells) under conditions of suboptimal Ag stimulation and/or inflammation in the presence of TGF-β. Mice expressing catalytically inactive p110δ (p110δ^D910A/D910A^) show increased thymic Treg cell development (15), possibly through enhanced Foxo transcription factor activity, which is required for Foxp3 expression and Treg cell function (16, 17). Treg cells also express higher levels of the PTEN and PHLPP phosphatases compared with conventional T (Tconv) cells, and deletion of these phosphatases lead to Treg cell destabilization and loss of function (18–20). In addition, withdrawal of TCR signaling and/or inhibition of the PI3K/AKT/mTOR pathway 18 h after T cell activation results in spontaneous Treg cell induction in vitro (induced Treg [iTreg]) (21). In contrast, work from our laboratory shows that despite the increase in Treg cell numbers in the thymus of p110δ^D910A/D910A^ mice, pTreg cell numbers are reduced (15), whereas PI3Kδ hyperactivation in p110δ^E1020K/WT^ mice results in increased pTreg cell numbers (22). Treg cells from p110δ^D910A/D910A^ mice also have impaired suppressive function in vitro, produce less IL-10, and express lower levels of CD38, a marker associated with superior suppressive function (15, 23).

PI3Kδ signaling is also required for optimal Treg cell function in vivo. Kinase-dead p110δ^D910A/D910A^ mice develop spontaneous colitis, elicited by gut microflora including *Helicobacter pylori*, which can be prevented by breeding mice under pathogen-free conditions (9). In addition, p110δ^D910A/D910A^ mice are resistant to *Leishmania major* infection despite attenuated Th1 cell responses as a result of compromised Treg cell expansion and homing to sites of infection (24). A similar mechanism also confers tumor resistance to p110δ^D910A/D910A^ mice and mice with a Treg cell–conditional p110δ deletion (25). Importantly, patients treated with the selective PI3Kδ inhibitor (idelalisib) suffer severe side effects, including autoinflammatory hepatotoxicity and colitis, which correlates with reduced peripheral blood Treg cell frequency and suppressive function (26, 27). Contrary to our expectations, mice with a Treg cell–conditional p110δ deletion did not show obvious signs of spontaneous autoimmunity or inflammation under pathogen-free conditions (25).

We considered the possibility that autoimmunity may develop in response to an inflammatory challenge in Treg cell–conditional p110δ-deficient mice. In addition, the other class I PI3K catalytic subunit isoforms may also play a role in Treg cell development and function, altering the requirement for signaling through PI3Kδ under specific conditions.

PI3Kδ and PI3Kγ act synergistically in thymic T cell development, and deletion/inactivation of both p110δ and p110γ isoforms results in T cell lymphopenia (28). However, mice with a deletion of p110γ in addition to p110δ inactivation have higher frequencies of pTreg cells with partially reduced function as measured by in vitro suppression assay (29). More recently, PI3Kδ inhibition but not concurrent p110γ deficiency was also shown to detrimentally affect Treg cell function in a mouse allograft transplantation model (30). We have previously demonstrated that PI3Kα can compensate for the loss of PI3Kδ function during early B cell development, with loss of both p110α and p110δ resulting in a profound block at the pre–B cell stage, but deletion of p110α in addition to p110δ in T cells did not result in an obviously altered phenotype (7). More recently, T cell–specific deletion of p110α was shown to enhance effector T cell function and reduce Treg cell expansion, resulting in delayed disease progression in a B16 melanoma model (31). This stands in contrast with an earlier study by Sauer et al. (21), showing that isoform-selective inhibitors of PI3Kα can promote in vitro iTreg differentiation to a greater extent than PI3Kδ or PI3Kγ inhibition. These studies highlight that the requirement of Treg cells for PI3K signaling may depend on specific activation conditions, and we considered that PI3Kα and PI3Kδ may have differential roles in Treg cell induction, maintenance, and function.

In this study, we show that, contrary to previously published data (32), genetic or pharmacologic inhibition of PI3Kδ signaling does not alter disease progression in an experimental autoimmune encephalitis (EAE) mouse model, possibly because of a simultaneous inhibitory effect on both T effector and Treg cell responses. By using mice with a Treg cell–conditional deletion of p110α, p110δ, or both isoforms, we show that PI3Kδ is the main isoform involved in maintaining a normal Treg cell phenotype, with partial compensation from PI3Kα for the loss of PI3Kδ activity. However, simultaneous deletion of both p110α and p110δ isoforms resulted in the loss of in vitro Treg cell suppressive function, lymphoproliferation and significantly increased EAE disease severity. Importantly, simultaneous loss of p110α and p110δ leads to spontaneous neuronal inflammation (neuropathy) and hind limb paresis in naive mice. These data indicate that PI3Kα can act synergistically with PI3Kδ to regulate Treg cell function under inflammatory conditions.

## Materials and Methods

### Mice

All breeding and in vivo procedures were carried out in accordance with the U.K. Home Office regulations (Animals [Scientific procedures] Act 1986) with approval from the Babraham Institute Animal Welfare and Ethics Review Body. Mice were maintained in individually ventilated cages under specific pathogen-free conditions at the Babraham Institute’s Biological Services Unit. We used male and female animals aged between 8 and 15 wk in all experiments.

PI3Kδ kinase dead (p110δ^D910A/D910A^) were as previously described (9). For in vitro experiments, age- and sex-matched C57BL/6 mice from the Babraham Institute breeding colony were used as controls in experiments with p110δ^D910A/D910A^ mice, and matched cohoused wild-type littermate controls were used for in vivo experiments. Foxp3^YFP-Cre^ (FYC-WT) mice (33) were obtained from Alexander Rudensky (Sloan Kettering Institute) and rederived into the Babraham Institute Biological Services Unit. FYC-WT mice were crossed to p110α^flox/flox^ mice (34) and p110δ^flox/flox^ mice (35) to delete p110α (FYC-p110α^fl^), p110δ (FYC-p110δ^fl^), or both isoforms simultaneously (FYC-p110α^fl^δ^fl^). Homozygous mice were used in all experiments. Treg cell–specific deletion of p110δ and/or p110α was confirmed by Western blot of cell lysates from sorted YFP^+^ Treg and YFP^−^ Tconv cell populations (Supplemental Fig. 1).

#### Summary of mouse lines.

The following mouse lines were used in this study. 

p110δ^D910A/D910A^: homozygous PI3Kδ kinase dead.

FYC-WT: Foxp3^YFP-Cre^ × wild-type p110α × wild-type p110δ.

FYC-p110α^fl^: Foxp3^YFP-Cre^ × p110α^flox/flox^.

FYC-p110δ^fl^: Foxp3^YFP-Cre^ × p110δ^flox/flox^.

FYC-p110α^fl^δ^fl^: Foxp3^YFP-Cre^ × p110α^flox/flox^ × p110δ^flox/flox^.

(Foxp3^YFP-Cre^ is X-linked; hence females were homozygous, males hemizygous).

### Buffers and media

#### Cell staining buffer.

Cells were suspended in PBS/2% FCS/0.05% NaN_3_ before being incubated with Abs prior to flow cytometry.

#### T cell medium.

T cells were maintained and stimulated in RPMI-1640 (Life Technologies) with 5% v/v FCS, 0.005% v/v 2-ME (Sigma), 1% v/v penicillin streptomycin (Life Technologies), 2 mM l-glutamine (Life Technologies), and 1% v/v 1 M HEPES.

#### T cell isolation buffer.

T cells were resuspended in PBS/1.25 mM EDTA/1% FCS before magnetic sorting.

#### Cell lysis buffer.

The 2× lysis buffer (100 mM HEPES, 300 mM NaCl, 20 mM NaF, and 20 mM iodoacetamide) was diluted 1:1 in distilled water and one Complete Mini Protease Inhibitor Cocktail Tablet (Roche) and 100 μl NP40 (BD Horizon) was added per 10 ml of buffer.

### Preparation of single cell suspensions from mouse tissues

Mice were culled by CO_2_ inhalation followed by cervical dislocation; dissected tissues were kept on ice while processed. Single cell suspensions were prepared from spleens, thymi, and lymph nodes by pushing the tissue through 40-μm cell strainers (BD Biosciences) using a syringe plunger. The cell suspensions were washed once with 5 ml cold PBS, and RBCs in spleen samples were lysed using hypotonic ammonium chloride RBC lysis buffer (Sigma), according to the manufacturer’s instructions. Spinal cord samples were prepared by dissecting the cord tissue from the vertebrae and pushing the tissue through 70-μm cell strainers using a syringe plunger. Cells were washed once in 5 ml cold PBS and collected by centrifugation. The cell pellets were resuspended in 37.5% isotonic Percoll (Sigma) at room temperature and centrifuged at 650 × *g* for 20 min with low acceleration and brake settings. The supernatant containing myelin and tissue debris was removed, and the cell pellets were washed twice by centrifugation in cold PBS. Cells were then resuspended at 1–3 × 10^6^ cells per sample and stained for flow cytometry as described below.

### T cell isolation

Negative selection of CD4^+^ T cells from peripheral lymph node cell suspensions were performed by immunomagnetic selection. All incubation and wash steps were performed in T cell isolation buffer unless otherwise indicated. Cells were resuspended at 1 × 10^8^ cells/ml, and FITC-conjugated Abs against mouse MHC class II, CD25, B220, CD8a, CD49b, and CD11b were added at a final dilution of 1:500 followed by incubation for 30 min at 4°C. Negative selection was performed using anti-FITC Microbeads (Miltenyi Biotec) and LS Magnetic Columns (Miltenyi Biotec), according to the manufacturer’s instructions, and unlabeled cells in the flow through were collected. Regular purity checks by flow cytometry confirmed enrichment to >95% CD4^+^ T cells.

### T cell differentiation assay

Isolated CD4^+^ T cells were stained with CFSE at a final concentration of 1 μM (Sigma) for 7 min at room temperature, resuspended at 2 × 10^6^ cells/ml in T cell culture medium, and transferred to anti-CD3 (1 μg/ml) and anti-CD28 (2 μg/ml)–coated flat-bottom 96-well plates. T cell culture medium supplemented with cytokines and blocking Abs was prepared as follows: Th17: TGF-β at 1 ng/ml, IL-6 at 20 ng/ml, IL-23 at 10 ng/ml, IL-1b at 10 ng/ml, anti-mouse IL-4 at 5 μg/ml, and anti-mouse IFN-γ at 10 μg/ml; Th1: IL-12 at 4 ng/ml, IL-2 at 20 ng/ml, and anti–IL-4 at 5 μg/ml; and Treg cell: TGF-β at 10 ng/ml, IL-2 at 20 ng/ml, anti-mouse IL-4 at 5 μg/ml, and anti-mouse IFN-γ at 10 μg/ml. Cells were cultured for 72 h, when half the culture medium was replaced with fresh medium containing replacement cytokines but not blocking Abs and cultured for a further 48 h. For detection of IFN-γ– and IL-17–producing cells, brefeldin A was added 3 h before collecting the cells and staining for flow cytometry.

### T cell suppression assay

YFP^+^ Treg cells were isolated by sorting lymph node samples pre-enriched for CD25^+^ cells using a mouse CD25 MicroBead Kit (Miltenyi Biotec) on a FACSAria (BD Biosciences). CD4^+^CD25^−^ Tconv cells were isolated from the CD25^−^ fraction obtained from the same samples by negative selection using FITC-conjugated Abs and anti-FITC Microbeads (Miltenyi Biotec) as described above (*T cell isolation*). Treg cells were cocultured in known ratios with 1 × 10^5^ Tconv cells per well in 96-well round-bottom Nunclon plates (Nunc) and stimulated with 2 × 10^4^ anti-CD3/anti-CD28–coated Dyna Beads (Dynal). After 96-h incubation at 37°C in an atmosphere of 5% CO_2_, proliferation was measured by [^3^H]thymidine incorporation. [^3^H]thymidine was added at 0.5 μl per well, and plates were incubated for 6 h before cells were harvested to UNIFILTER Plates (PerkinElmer) using a Tomtec 96 Harvester. Collection plates were air dried overnight and 30 μl MicroScint-20 (PerkinElmer) added to each well. A TopCount Scintillation Counter (PerkinElmer) was then used to read the relative incorporation of [^3^H]thymidine in each well.

### Treg cell activation assay for pAkt analysis

Live cells were isolated from lymph node single cell suspensions by layering over Lympholyte-M (Cedarlane Laboratories), according to the manufacturer’s instructions. Cells were counted and resuspended in T cell medium at 1 × 10^8^ cells/ml. Cells were incubated for 1 h on ice with biotinylated anti-mouse CD3 (clone 145-2C11) and anti-mouse ICOS (clone 7E-17G9) (eBioscience) at 1 and 2 μg/ml, respectively. For Na_3_VO_4_ stimulation, cells were incubated for 1 h on ice without Abs. Cells were washed twice by centrifugation, resuspended at 3 × 10^7^ cells/ml in T cell medium, and aliquoted into microcentrifuge tubes at 100 μl per condition. For assessing the effects of PI3K inhibitors, cells were treated with idelalisib (100 nM), alpelisib (500 nM), or DMSO control as required, incubating for 15 min in a heat block at 37°C prior to stimulation. Where PI3K inhibitor treatment was not required, cells were incubated for 2 min at 37°C prior to stimulation. For anti-CD3/anti-ICOS stimulation, streptavidin (Jackson ImmunoResearch) was added at a final concentration of 10 μg/ml; alternatively, 0.5 mM Na_3_VO_4_ was added. The reaction was stopped after 5 min (anti-CD3/anti-ICOS) or 1 min (Na_3_VO_4_) by adding 300 μl ice-cold 4% paraformaldehyde (Intracellular Fixation Buffer; BioLegend). Cells were incubated 15 min on ice, washed in Foxp3 Transcription Factor Wash Buffer (Invitrogen), and then stained with Abs against CD4, CD8, CD25, Foxp3 and pAKT for 1 h at room temperature in Foxp3 Transcription Factor Wash Buffer (Invitrogen). Cells were then washed twice in PBS before analysis by flow cytometry.

### Flow cytometry

Single cell suspensions (1–3 × 10^6^ cells per sample) were stained for flow cytometry. For detecting cell surface Ags, cells were washed once and incubated for 30 min at 4°C with an Ab master mix prepared in cell staining buffer (PBS/2% FCS/0.05% NaN_3_). Cells were washed once and fixed using a 4% paraformaldehyde solution. When biotinylated Abs were used, cells were stained for 15 min at 4°C with fluorophore-conjugated streptavidin prior to fixation. For the detection of intracellular cytokines, cells were resuspended in T cell culture medium and stimulated with 50 ng/ml phorbol dibutyrate (Tocris Bioscience), 1 μM ionomycin (Sigma), and brefeldin A (eBioscience) for 4–6 h at 37°C. Intracellular staining for the detection of Foxp3 was performed using the eBioscience Foxp3/Transcription Factor Staining Buffer Set, according to the manufacturer’s instructions. Intracellular staining for the detection of cytokines were performed using the BioLegend Intracellular Staining Permeabilization/Wash Buffer, according to the manufacturer’s instructions. Stained samples were analyzed using BD LSRIIFortessa/Fortessa5, Cytek Aurora, or Life Technologies Attune Flow Cytometers; data analysis was performed in FlowJo (Tree Star). Flow cytometry Abs are summarized in Table I.

### EAE model

EAE was induced by s.c. injection of 250 μg myelin oligodendrocyte glycoprotein peptide aa 35–55 ([MOG_(35–55)_] MEVGWYRSPFSRVVHLYRNGK) (thinkpeptides) emulsified in CFA (Sigma) across two sites at the tail base/flank. Mice also received an i.p. injection of 200 ng pertussis toxin (*Bordetella pertussis)* (Sigma) in sterile PBS at the time of MOG_(35–55)_ immunization and again 48 h postimmunization. The PI3Kδ inhibitor IC87114 was provided by Jonathan Clark (Babraham Institute) and given twice daily by oral gavage at 30 mg/kg as a suspension in 0.5% carboxymethylcellulose in water. This dosing strategy was previously shown to modulate T cell–mediated immune responses in vivo (36). Treatment was started 24 h before EAE induction and maintained for the duration of the study. Mice were weighed once weekly until weight loss was detected and then daily until the weight remained stable for three consecutive days. All mice were observed daily for 25–35 d and scored according to the criteria in Table II when clinical signs appeared. Scoring was performed by at least two individuals who were blinded to genotype for each study. Signs of ascending paralysis usually appeared from 7 d onwards. At this stage, mice were housed on extra absorbent bedding (α-dri), and soft food and water was provided on the cage floor. Animals were culled by a schedule 1 method when showing a score of five or had difficulty moving around the cage on two consecutive days or when they lost 25% or more of their body weight. On rare occasions, mice were found dead or moribund at which stage, they were culled immediately and assigned an EAE score of 6. Peak disease severity was observed at 14–16 d post–MOG_(35–55)_ immunization, and animals were culled at this time to assess T cell infiltration into the CNS and draining lymph nodes.

### Western blot

YFP^+^ Treg cells were sorted on a BD FACSAria cell sorter from pooled lymph node samples preenriched for CD4^+^ cells by immunomagnetic negative selection, as described above. Cells were counted and suspended in 35 μl cell lysis buffer per 1 × 10^6^ cells before incubating on ice for 10 min. Samples were then centrifuged at 15,000 rpm at 4°C for 10 min. Supernatant was aliquoted, and 10 μl NuPAGE Running Buffer (Life Technologies) was added per 30 μl supernatant. One molar DTT-reducing agent (Life Technologies) was then added at a ratio of 1 in 50 to samples, which were heated at 70°C for 10 min. NuPAGE 4–12% Bis-Tris Gels were loaded with sample and run in NuPAGE SDS MOPS Running Buffer using the XCell SureLock Mini-Cell Electrophoresis System (Life Technologies) for 50 min at 200 V and 210 mA. Blotting pads and filter paper were soaked in transfer buffer (1× NuPAGE Transfer Buffer [Life Technologies], 10% methanol (VWR), and 1:1000 NuPAGE Antioxidant [Life Technologies]). PVDF membranes (GE Healthcare) were soaked for 1 min in methanol and rinsed in double distilled water (ddH_2_O). Proteins were transferred to the membrane at 30 V for 1 h using the XCell II Blot Module (Life Technologies) in accordance with manufacturer’s instructions. Blots were blocked for 1 h at room temperature in 5% w/v milk (Marvel Biotech) in TBST (150 mM NaCl [AnalaR], 50 mM Tris-HCl [Melford] [pH 7.6], plus 0.1% Tween 20). After rinsing in TBST, blots were probed overnight at 4°C with 1:100 anti-p110α (C73F8; Cell Signaling Technology) or 1:2000 anti-p110δ (Abcam) in TBST with 5% w/v BSA and 0.05% w/v NaN3. Blots were washed three times in TBST and then incubated for 1 h at room temperature with 1:25,000 HRP-conjugated goat anti-rabbit IgG (Dako) in 5% w/v milk in TBST. After three further washes, light signals were detected using ECL detection reagent and Hyperfilm (GE Healthcare), developed using a Compact ×4 developer (Xograph). To reprobe blots for proteins of similar size, blots were stripped by incubation with stripping buffer (2% SDS, 100 mM 2-ME, 50 mM Tris [Sigma] [pH 6.8]) at 50°C for 15–30 min. Membranes were washed three times in TBST before blocking and reprobing as described above.

### Histology

For histopathological review, formalin-fixed tissues were sent to ProPath (Hereford, U.K.) for paraffin embedding, sectioning, and H&E staining. Unstained slides were prepared for immunohistochemistry. To examine demyelination, samples embedded by ProPath were sent to the histopathology service at the Royal Veterinary College for Luxol Fast Blue staining.

#### Interpretation and scoring.

H&E-stained sections were initially screened unblinded for the presence of lesions. Pathological findings were then scored blindly on a semiquantitative scale ranging from 0 to 5, according to Table III (37).

### Statistics and data analysis

Data analysis was performed in Graphpad Prism. D’Agostino and Pearson or Shapiro–Wilk normality tests were performed for all datasets. For datasets following a Gaussian distribution, Student *t* test with Welch correction was used to compare two groups; one-way ANOVA with Tukey multiple comparisons test was used where three or more groups were compared. Datasets not following a Gaussian distribution were analyzed using the Mann–Whitney *U* test for comparing two groups or the Kruskal–Wallis test with Dunn’s multiple comparison test for three or more groups. Statistical significance is indicated in the figures as follows: *p ≤ 0.05, **p ≤ 0.01, ***p ≤ 0.001, and ****p ≤ 0.001. For EAE studies, disease scores for each timepoint were compared by one-way ANOVA, and survival curves were compared using the Gehan–Breslow–Wilcoxon test. In vivo studies were blinded as follows: animals were genotyped and allocated to age- and sex-matched study groups. Mice were identified by implanted microchips so that the identity was not apparent to the operator. MOG_(35–55)_ immunizations and EAE scoring were done without knowledge of genotypes, and genotypes were revealed only at the end of the study for analysis. For all studies, monitoring was shared between one to two researchers and two technicians not responsible for the design, analysis, or interpretation of the study.

## Results

### Simultaneous loss of PI3Kδ and PI3Kα signaling increases EAE disease severity

**FIGURE 1.**
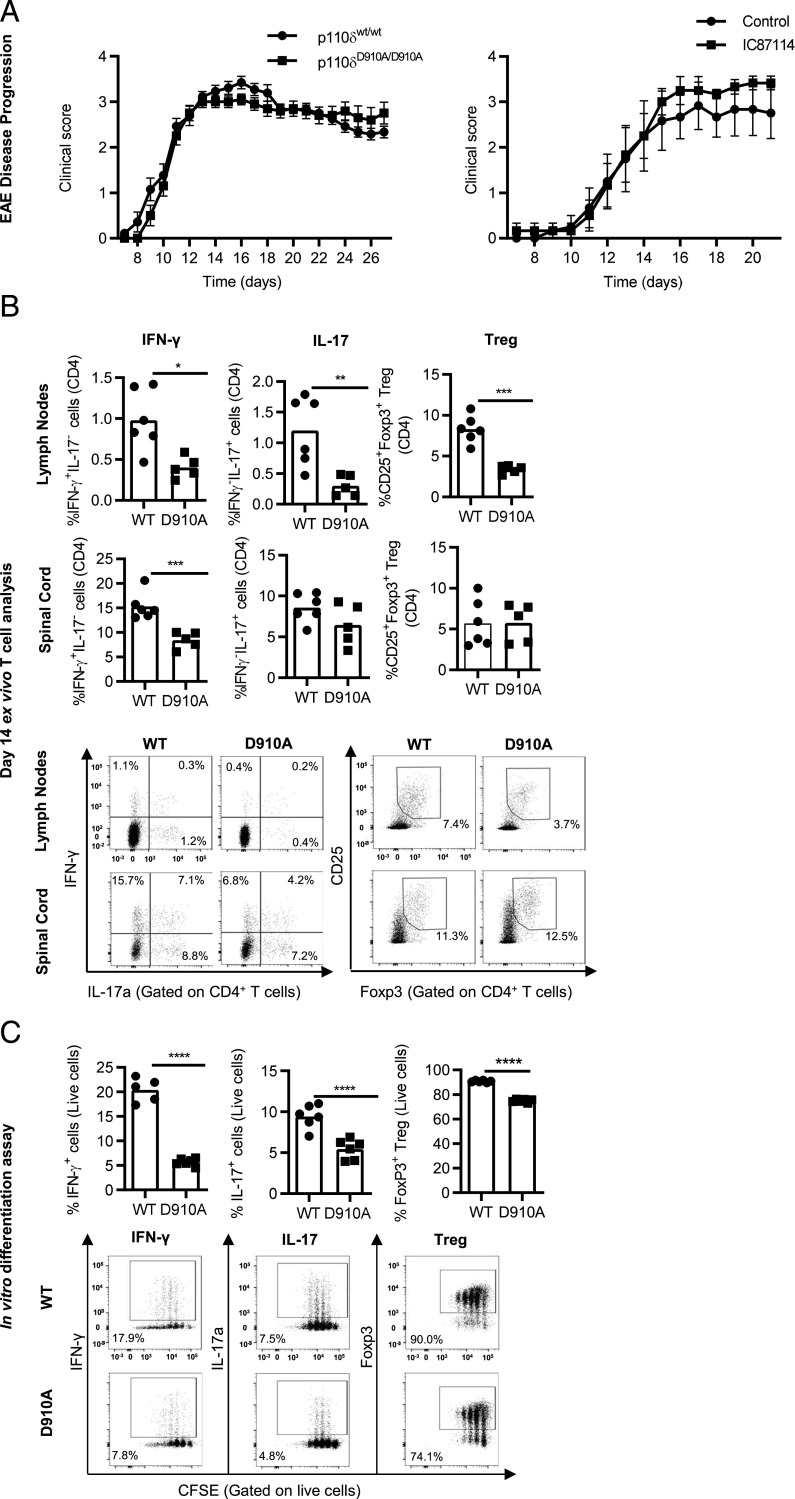
PI3Kδ inhibition does not influence EAE disease progression because of attenuation of both proinflammatory and Treg cell responses. EAE was induced in mice by s.c. injection of MOG_35–55_ peptide in CFA, followed by two doses of pertussis toxin at 0 and 48 h postimmunization. (**A**) No difference in clinical score was observed when comparing wild-type and p110δ^D910A/D910A^ littermate controls or when comparing C57BL/6 mice receiving twice daily doses of IC87114 (30 mg/kg) to mice receiving vehicle control by oral gavage. Mean ± SEM, *n* = 12; results representative of at least two independent experiments. (**B**) We found reduced proportions of IFN-γ–producing CD4^+^ T cells in the draining lymph nodes and spinal cords of p110δ^D910A/D910A^ mice compared with wild-type controls at 14 d post–EAE induction. We also found a significant decrease in IL-17–producing CD4^+^ T cells and Foxp3^+^ Treg cells in the draining lymph nodes, but not spinal cord at 14 d post–EAE induction. CD4^+^ T cells were identified by flow cytometry by excluding debris based on forward and side scatter and then gating on single cells, followed by live CD45^+^ cells and then CD3^+^ T cells and then gating on CD4^+^ T cells. (**C**) Naive T cells were isolated from p110δ^D910A/D910A^ and C57BL/6 mice and cultured for 5 d in the presence of anti-CD3/anti-CD28 stimulation and polarizing cytokines: Th1, 10 μg/ml anti–IL-4, 4 ng/ml IL-12; Th17, 10 μg/ml anti–IL-4 and anti–IFN-γ, 1 ng/ml TGF-β, 20 ng/ml IL-6, and 10 ng/ml IL-23 and IL-1β; Treg cells, 10 μg/ml anti-CD4 and anti–IFN-γ, 10 ng/ml TGF-β, and 20 ng/ml IL-2. Cells isolated from p110δ^D910A/D910A^ mice maintained the potential for differentiation into Th1, Th17, and Treg cells, but differentiation was less efficient than wild-type cells. Live cells were identified by flow cytometry by excluding debris based on forward and side scatter and then gating on single cells, followed by live cells. Mean + Scatter, *n* = 5–6; results representative of at least two independent experiments. *p < 0.05, **p < 0.01, ***p < 0.001, ****p < 0.001.

Inactivation of PI3Kδ by point mutation (p110δ^D910A/D910A^) or by administration of the PI3Kδ-selective inhibitor IC87114 did not alter the progression of MOG_(35–55)_–induced EAE compared with wild-type or vehicle-treated mice (Fig. 1A). The analysis of CD4^+^ T cells present in the draining lymph nodes and spinal cord of p110δ^D910A/D910A^ mice during peak disease severity (day 15) showed reduced proportions of IFN-γ– and IL-17–producing cells compared with wild-type littermate controls (Fig. 1B). However, proportions of protective CD25^+^Foxp3^+^ Treg cells were also decreased in the draining lymph nodes of MOG-immunized mice (Fig. 1B). Furthermore, naive T cells from p110δ^D910A/D910A^ mice were less able to differentiate into Th1, Th17, or Treg cells in vitro compared with wild-type T cells (Fig. 1C). In addition, previous studies from our laboratory and others have demonstrated reduced Treg cell function in p110δ^D910A/D910A^ mice in vitro and in vivo in the context of autoimmunity, infection, and cancer. Therefore, we considered that the reduction in Treg cell numbers and/or function may counteract the potential benefit of reduced Th1 and Th17 cells (Fig. 1B).

**Table I.** Flow cytometry Abs

Ag	Fluorochrome	Clone	Concentration (μg/ml)	Supplier
CD3	FITC	145-2C11	1	eBioscience
CD4	V500; PerCPcy5.5	RM4-5	1	BD Horizon
CD8a	eF450; APC-Cy7	53-6.7	1	eBioscience
CD25	PE; BV605	PC61	1	BioLegend
CD38	APC	90	0.5	BioLegend
ICOS (CD278)	PE-cy5	7E.17G9	1	eBioscience
CD103	PE	2E7	1	eBioscience
Helios	Pacific Blue	22F6	0.5	BioLegend
NRP1 (CD304)	PE	3DS304M	1	eBioscience
IL-17A	Alexa647	eBio17B7	1	eBioscience
IFN-γ	PE-Cy7	XMG1.2	1	eBioscience
Foxp3	Alexa647; eF450	FJK-16S	2	eBioscience
B220	APC-cy7	RA3-6B2	1	BioLegend
CD45	PerCPcy5.5	30-F11	1	eBioscience
pAKT(ser473)	Alexa647	D9E	2.5	Cell Signaling Technology
Viability dyes	eF450; eF780	none	1:5000	eBioscience

**Table II.** EAE scoring criteria

Score	Observation
0	Normal
0.5	Weak tail
1	Flaccid tail
1.5	Flaccid tail, hind limb weakness, normal gait, and righting reflex
2	Flaccid tail, hind limb weakness, abnormal gait, and/or righting reflex
2.5	Flaccid tail, hind limb weakness, and absent righting reflex
3	Flaccid tail and partial hind limb paralysis
4	Flaccid tail and total hind limb paralysis
5	Flaccid tail, total hind limb paralysis, and partial fore limb paralysis
6	Moribund or found dead

**Table III.** Histopathology grading scheme

Score	Observation
0	Absent
1	Minimal
2	Slight
3	Moderate
4	Moderately severe
5	Severe

Grading scheme based on those presented in Shackelford et al. (37).

**FIGURE 2.**
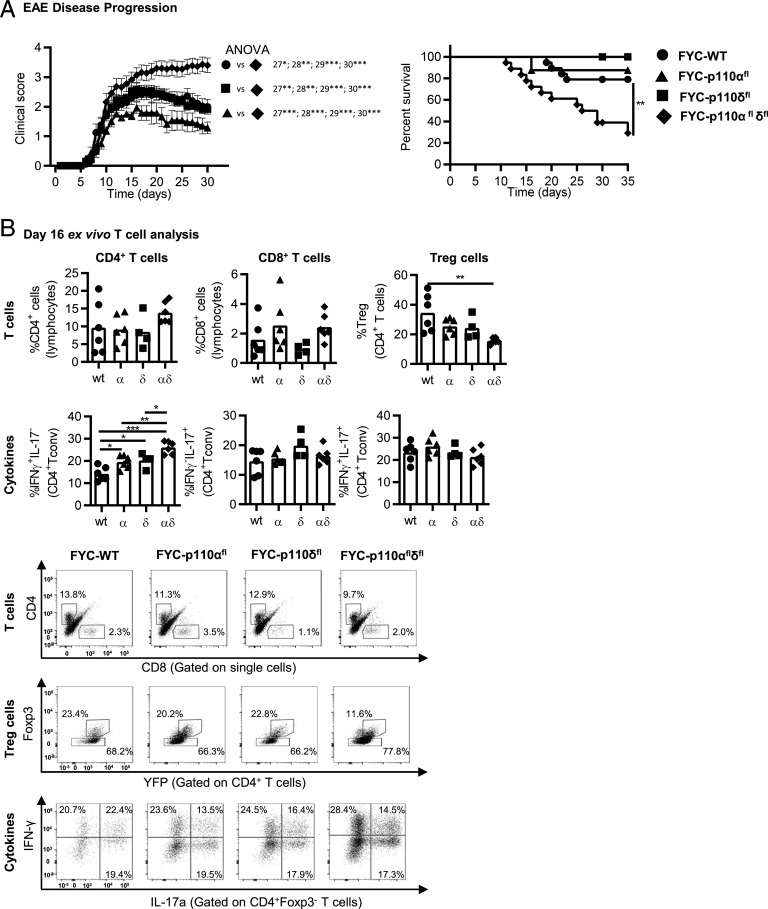
Simultaneous loss of p110α and p110δ in Treg cells promotes EAE disease development. (**A**) EAE was induced in mice by s.c. injection of MOG_35–55_ peptide in CFA, followed by two doses of pertussis toxin at 0 and 48 h postimmunization. Treg cell–restricted loss of both p110α and p110δ leads to increased disease scores and mortality, whereas individual loss of p110α or p110δ does not significantly affect disease outcome compared with wild-type controls. Survival curves were analyzed using the Gehan–Breslow–Wilcoxon test, and disease scores were compared by one-way ANOVA for each timepoint, statistical significance indicated from day 27 onwards. Mean ± SEM, *n* = 8–12, combined results from two independent experiments. (**B**) The exacerbated disease phenotype in response to the loss of both p110α and p110δ is associated with reduced Treg cell infiltration into the spinal cord. Among the conventional CD4^+^ T cells present in the spinal cord, cells expressing IFN-γ are increased compared with control mice. Single cells were identified by flow cytometry by excluding debris based on forward and side scatter and then gating on single cells. Mean + Scatter, *n* = 5–6; results representative of at least two independent experiments. *p < 0.05, **p < 0.01, ***p < 0.001.

Contrary to our expectations, Treg cell–specific deletion of p110δ did not affect MOG_(35–55)_–induced EAE progression (Fig. 2A). This result was surprising as we have previously shown that Treg cell–specific deletion of p110δ renders mice resistant to tumor growth as a result of defective Treg cell function. We considered that deleting the p110δ protein, instead of inactivation by point mutation or an isoform-selective PI3Kδ inhibitor, could allow for compensation by p110α, which has also been implicated in Treg cell function (21, 31). Indeed, simultaneous deletion of p110α and p110δ led to significantly increased peak disease severity, increased mortality (Fig. 2A), and increased CNS inflammation at day 16, as assessed by histopathological scoring of H&E–stained spinal cord sections (Supplemental Fig. 2). Analysis of CD4^+^ T cells infiltrating the spinal cord at peak disease severity (day 16) showed that there was a trend toward increased proportions of total CD4^+^ T cells in FYC-p110α^fl^δ^fl^ mice and that this was associated with a significant reduction in the proportion of CD4^+^Foxp3^+^ Treg cells (Fig. 2B). In addition, the proportion of IFN-γ–producing CD4^+^Foxp3^−^ Tconv cells were increased in FYC-p110α^fl^, FYC-p110δ^fl^, and FYC-p110α^fl^δ^fl^ mice compared with FYC-WT controls. Simultaneous deletion of p110α and p110δ in Treg cells resulted in significantly higher proportions of IFN-γ–producing CD4^+^ Tconv cells in the spinal cord compared with Treg cell–conditional loss of either p110α or p110δ on its own (Fig. 2B). These data indicate that p110α can compensate for the loss of p110δ in maintaining the ability of Treg cells to restrict effector T cell responses in the CNS.

### Differential roles of PI3Kδ and PI3Kα in Treg cell development and function

**FIGURE 3.**
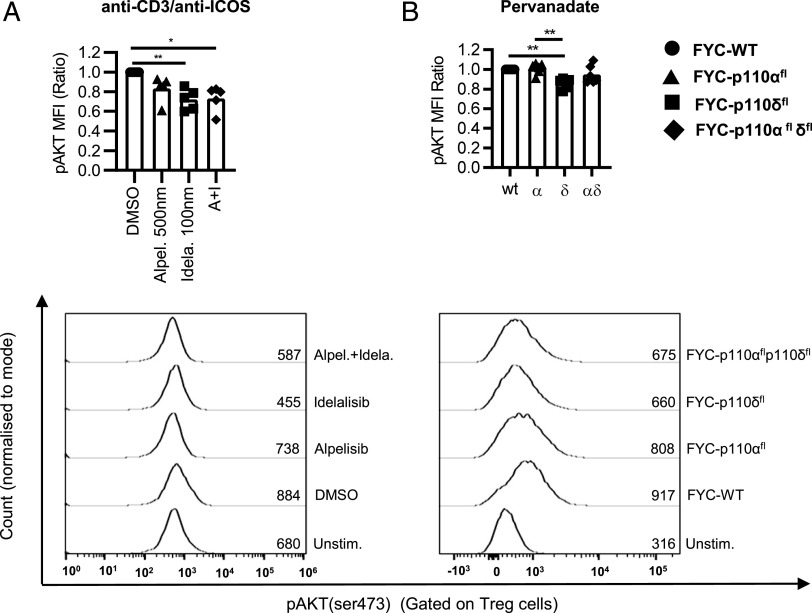
PI3Kδ is the major isoform involved in AKT phosphorylation in Treg cells following in vitro stimulation. (**A**) Lymph node single cell suspensions from naive wild-type mice were incubated with biotinylated anti-CD3 and anti-ICOS, and cells were stimulated for 5 min at 37°C by cross-linking with streptavidin in the presence of selective inhibitors for p110α (alpelisib, 500 nM), p110δ (idelalisib, 100 nM), or both combined (A+I). Unstimulated samples (US) were treated with DMSO and incubated for 5 min without the addition of streptavidin. Selective inhibition of p110δ and combined inhibition resulted in significantly lower AKT phosphorylation in Treg cells as measured by flow cytometry. (**B**) Lymph node single cell suspensions from naive FYC-WT, FYC-p110α^fl^, FYC-p110δ^fl^, or FYC-p110α^fl^δ^fl^ mice were stimulated with pervanadate for 1 min at 37°C. Treg cell–specific deletion of p110δ and combined p110α/δ deletion resulted in significantly lower AKT phosphorylation in Treg cells as measured by flow cytometry. Treg cells were identified by flow cytometry by excluding debris based on forward and side scatter and then gating on single cells, followed by CD4^+^ cells, and then gating on CD25^+^Foxp3^+^ Treg cells. *n* = 5; results from five independent experiments combined. *p < 0.05, **p < 0.01.

**FIGURE 4.**
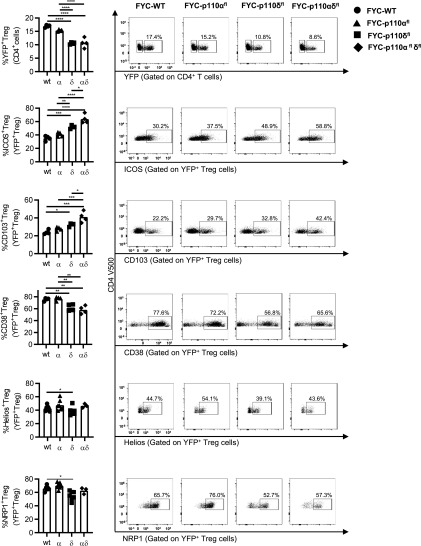
PI3Kδ is required for the maintenance of normal Treg cell proportions and phenotype. Spleen single cell suspensions from naive FYC-WT, FYC-p110α^fl^, FYC-p110δ^fl^, and FYC-p110α^fl^δ^fl^ mice were stained with Treg cell functional and phenotypic markers and analyzed by flow cytometry. Treg cell–specific deletion of p110δ results in reduced Treg cell proportions in the spleen and in reduced proportions of Treg cells expressing CD38, Helios, and NRP1, whereas additional deletion of p110α does not have an additive effect. Deletion of p110δ in Treg cells also results in increased expression of ICOS and CD103, and additional deletion of p110α further leads to a significant increase in expression compared with p110δ deletion alone. CD4^+^ T cells were identified by flow cytometry by excluding debris based on forward and side scatter and then gating on single cells, followed by excluding B cells based on B220 expression and then gating on CD4^+^ cells. *n* = 4–6; results representative of at least two independent experiments. *p < 0.05, **p < 0.01, ***p < 0.001, ****p < 0.001.

**FIGURE 5.**
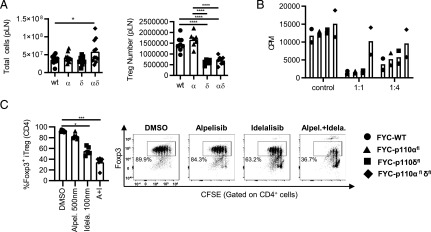
Simultaneous deletion of both p110δ and p110α in Treg cells results in reduced suppressive function and defective Treg cell differentiation in vitro. (**A**) Total lymph node cell numbers were determined by automatic counting (CASY counter), and YFP^+^ Treg cell numbers were determined by flow cytometry (see gating strategy in Fig. 4). Combined deletion of p110δ and p110α in Treg cells results in increased cellularity of the peripheral lymph nodes, whereas deletion of p110δ and combined p110α/δ deletion lead to a similar reduction in Treg cell number. (**B**) CD4^+^YFP^+^ Treg cells were sorted from FYC-WT, FYC-p110α^fl^, FYC-p110δ^fl^, and FYC-p110α^fl^δ^fl^ mice and cocultured in known ratios with FYC-WT Tconv cells for 96 h. Cocultures were stimulated with anti-CD3– and anti-CD28–coated beads, and proliferation was measured by [^3^H]thymidine incorporation. Combined deletion of p110α and p110δ results in a suppressive defect at ratios of 1:1 and 1:4. Results are representative of two independent experiments. (**C**) Naive T cells were isolated from wild-type mice and cultured for 5 d in the presence of anti-CD3/anti-CD28/ml IL-2 in the presence of selective PI3K inhibitors under Treg cell–polarizing conditions (10 μg/ml anti-CD4 and anti–IFN-γ, 10 ng/ml TGF-β, and 20 ng/ml IL-2). PI3Kδ inhibitor: idelalisib, 100 nM; PI3Kα alpelisib: 500 nM; or both inhibitors combined (A+I). PI3Kα inhibition does not have a significant effect on in vitro Treg cell differentiation but exacerbates the effect of PI3Kδ inhibition. CD4^+^ cells were identified by flow cytometry by excluding debris based on forward and side scatter and then gating on single cells, followed by live CD4^+^ cells. *n* = 5–6; results are representative from at least two independent experiments. *p < 0.05, **p < 0.01, ***p < 0.001, ****p < 0.001.

In vitro stimulation of T cells from naive mice showed that Akt phosphorylation was dependent on signaling through p110δ and that p110α did not contribute or compensate for inactivation or loss of p110δ (Fig. 3A, 3B). However, analysis of Treg cell functional and phenotypic marker expression in the spleens of naive mice revealed differential roles for p110α and p110δ. Treg cell–specific deletion of p110δ and combined p110α/δ deletion resulted in a comparable decrease in Treg cell proportions as well as the proportion of CD38-expressing Treg cells consistent with our previous findings in p110δ^D910A/D910A^ mice (15, 23). Deletion of p110δ resulted in slightly fewer Treg cells expressing Helios and neuropilin-1 (NRP1), but these markers were apparently not affected by p110α deletion (Fig. 4). ICOS and CD103 expression were not affected by p110α deletion but were significantly increased in response to the loss of p110δ in Treg cells. This effect was exacerbated by the additional loss of p110α, indicating that p110α can partially compensate for the loss of p110δ in this context (Fig. 4). These data show that p110α can contribute to some phenotypic changes primarily controlled by p110δ. However, simultaneous loss of p110α and p110δ in Treg cells, but not deletion of either isoform on its own, resulted in spontaneous lymphoid hyperplasia in some mice (Fig. 5A) and reduced suppressive capacity in vitro (Fig. 5B). In addition, the combined inhibition of p110δ and p110α using isoform-selective PI3K inhibitors resulted in a profound reduction in the proportion of differentiated iTreg cells (Fig. 5C). We previously found that p110δ^D910A/D910A^ mice have increased numbers of Treg cells in the thymus, which could reflect impaired negative selection of autoreactive T cells that are instead induced to express Foxp3 (15). However, Foxp3^YFP-Cre^–mediated deletion of p110α or p110δ did not result in altered thymic Treg cell numbers (Supplemental Fig. 3A), perhaps reflecting a requirement for PI3K activity during early thymic Treg cell differentiation (before Foxp3 is expressed) rather than for maintenance of Treg cell numbers. This is supported by the observation that TGF-β–mediated induction of Foxp3^+^ Treg cells is impaired in naive CD4^+^ T cells from p110^D910A/D910A^ mice or in wild-type cells treated with a PI3Kδ inhibitor but not from FYC-p110α^fl^, FYC-p110δ^fl^, or FYC-p110α^fl^δ^fl^ mice (Supplemental Fig. 3B).

### Simultaneous loss of PI3Kδ and PI3Kα results in spontaneous pelvic limb paresis

**FIGURE 6.**
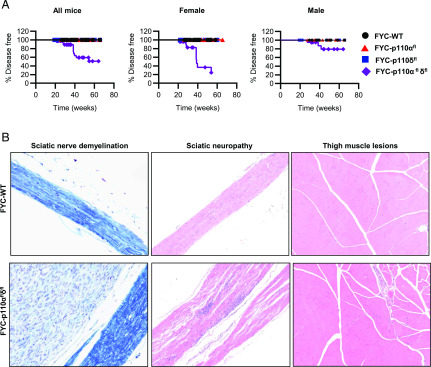
Simultaneous deletion of both p110δ and p110α in Treg cells results in spontaneous pelvic limb paresis. (**A**) The prevalence of pelvic limb gait abnormalities observed in the general, aged mouse population (>127 d) was recorded. Mice maintained for general tissue collection, mice aged specifically to examine pelvic limb paresis, and breeders are included in the data. Female mice deficient in both p110α and p110δ were more likely to develop spontaneous pelvic limb paresis. (**B**) This was associated with sciatic nerve demyelination as assessed by Luxol Fast Blue staining (images at original magnification ×20) as well as sciatic neuropathy and thigh muscle lesions as assessed by H&E staining (images at original magnification ×10).

We noticed the onset of pelvic limb gait abnormalities in older FYC-p110α^fl^δ^fl^ mice but not in FYC-p110α^fl^ or FYC-p110δ^fl^ mice. The earliest age of disease onset was 127 d. Therefore, we retrospectively compared the health records for all mice aged over 127 d and found that females were more susceptible, with 32.1% (9/28) of FYC-p110α^fl^δ^fl^ females developing hind limb paresis compared with 13.6% (3/22) of FYC-p110α^fl^δ^fl^ males. In contrast, none of the 34 FYC-WT mice, 36 FYC-p110α^fl^ mice, or 37 FYC-p110δ^fl^ mice analyzed showed gait abnormalities (Fig. 6A). Furthermore, histological examination of the sciatic nerve revealed demyelination and inflammatory lesions in clinically affected FYC-p110α^fl^δ^fl^ mice. Cellularity of the sciatic nerve was increased, and inflammatory cells were present within and around the nerve (Fig. 6B). These changes were indicative of nerve inflammation and degeneration and were not reported in age-matched clinically healthy FYC-p110α^fl^δ^fl^ mice or in age-matched individuals from any other genotype. In addition, FYC-p110α^fl^δ^fl^ mice exhibiting spontaneous pelvic limb paresis also showed significant lesions of the thigh muscle. These animals showed a small increase in the prevalence of degenerating and regenerating muscle fibers and, more prominently, inflammation around the nerves (Fig. 6B). These data, together with the increased susceptibility to MOG_35–55_–induced EAE, show that loss of both p110α and p110δ in Treg cells predisposes mice to autoimmune neuropathy. This observation indicates that the PI3Kα complex can functionally compensate for the loss of PI3Kδ activity in Treg cells.

## Discussion

Class I PI3K signaling impacts multiple aspects of the immune response, with the PI3Kα, PI3Kβ, PI3Kγ, and PI3Kδ complexes playing distinct roles in the development, proliferation, migration, and function of myeloid cells as well as B and T lymphocytes. Although several unresolved complexities remain, catalytic subunit isoform-specific inhibition of the class I PI3K signaling pathway has become a therapeutic option in the treatment of cancer, inflammatory conditions, and immunodeficiencies associated with PI3K hyperactivation (8, 26, 38–42). However, the sometimes-severe adverse effects associated with the PI3Kδ-specific inhibitor idelalisib underlines the importance of understanding the relative contribution of PI3K signaling in different immune cell subsets under steady-state and inflammatory conditions (26).

We found that PI3Kδ inhibition did not affect the progression or disease severity of T cell–mediated autoimmune inflammation in an EAE mouse model. This finding was unexpected and stands in contrast to a previous study showing that PI3Kδ inactivation could ameliorate disease severity through reduced Th17 cell–mediated inflammation (32). Although the reason for this discrepancy is not completely clear, small differences in the microbiome of experimental and control animals bred separately can affect the outcome of EAE disease progression. Therefore, we used littermate controls in all in vivo experiments, whereas Haylock-Jacobs et al. used wild-type C57BL/6 mice purchased from an external supplier. Although we found a significant reduction in Th17 and Th1 cells in response to PI3Kδ inhibition, we also saw a reduction in the numbers of Treg cells. Treg cell–mediated immune regulation is important in controlling autoimmune inflammation in this model (43–46). Therefore, it is possible that any potential benefit from an attenuated effector T cell response is balanced by a concomitant reduction in immune regulation. In fact, work from our laboratory showed that PI3Kδ inhibition can promote anti-tumor immune responses by restraining Treg cell responses to a greater extent compared with effector T cell responses (25). Consequently, we predicted that Treg cell–conditional deletion of p110δ would result in a more severe EAE disease phenotype but found that this was not the case, despite decreased pTreg cell proportions and numbers as well as reduced CD38-expressing Treg cells under steady-state conditions. These data mimic previous findings in p110δ^D910A/D910A^ mice and indicate that although PI3Kδ is the main isoform involved in the homeostatic regulation of Treg cells, other compensatory mechanisms may exist to preserve Treg cell function in response to the loss of PI3Kδ signaling. We explored the possibility that p110α could compensate for the loss of p110δ in the context of CNS inflammation.

Two studies indicate opposing roles for PI3Kα in Treg cell differentiation (21, 31). We found that conditional deletion of p110α in Treg cells did not alter EAE disease progression or Treg cell numbers and phenotype. However, deletion of p110α in addition to p110δ significantly increased the upregulation of ICOS and CD103 seen in response to the deletion of p110δ alone. ICOS is expressed on a subpopulation of highly suppressive Treg cells; however, signaling through ICOS in CD4^+^ T cells depend on PI3K activity (12, 31, 47, 48). It is possible that the upregulation of ICOS in response to the loss of PI3K activity is a consequence of disrupted PI3K-dependent negative feedback. A previous study showed that PI3Kα preferentially binds to phosphorylated ICOS peptides and to ICOS immunoprecipitates from activated T cells (49). This indicates a potential role for PI3Kα in maintaining ICOS-dependent Treg cell function in vivo and could explain why increased CNS inflammation and EAE severity is only observed when both p110δ and p110α are deleted in Treg cells. ICOS costimulation plays an important role in effector T cell responses and in maintaining B cell responses through T follicular helper cell generation, and ICOS/ICOSL inhibition ameliorates several autoimmune conditions, such as acute graft-versus-host disease, rheumatoid arthritis, and systemic lupus erythematosus (50). However, ICOS is also critical for the normal function of pTreg cells (51). ICOS-deficient mice developed more severe EAE; furthermore, ICOS blockade in the first 10 d after EAE induction lead to increased disease severity (52, 53). These studies did not differentiate between the role of ICOS in effector versus Treg cells, and it is possible that ICOS plays a specific role in Treg cell–mediated immune regulation in the CNS. Indeed, in NOD mice, ICOS deficiency protects against the spontaneous development of autoimmune type 1 diabetes but results in the development of neuromuscular autoimmunity associated with inflammatory cell infiltrates in the CNS (54, 55). This condition resembles the spontaneous hind limb paresis associated with sciatic nerve demyelination and inflammation we observed in FYC-p110α^fl^δ^fl^ mice (aged 32–81 wk). This was not observed in wild-type mice or in response to individual p110α or p110δ deletion in the same cohort. It is interesting to note that females were more susceptible than male mice as this is often the case in human autoimmune disorders and indicate that the mechanisms underlying this phenotype could be relevant to human disease (56). Spontaneous peripheral neuropathy has been described in females aged 99–100 wk from several mouse strains, including C57BL/6 mice (57). However, among 2930 48–78-wk-old C57BL/6 mice housed at the Babraham Institute breeding unit while the current study was in progress, only 12 cases of spontaneous hind limb paresis have been recorded, indicating that the prevalence of hind limb paresis among wild-type C57BL/6 mice housed under the same conditions as the FYC-p110α^fl^δ^fl^ mice is very low (0.4%). Therefore, we can conclude that the combined loss of p110α and p110δ increases susceptibility to CNS damage in the context of EAE and spontaneous peripheral nerve damage leading to paralysis or paresis, respectively.

These data indicate that although p110δ is the dominant PI3K isoform in Treg cells, p110α can compensate in part for the loss of p110δ in maintaining Treg cell function in the context of autoimmune inflammation. This is further supported by the observation that only combined Treg cell–conditional deletion of p110α and p110δ results in the failure to control Tconv cell proliferation in response to anti-CD3 and anti-CD28 stimulation in vitro. This effect is context dependent as the deletion of p110δ in Treg cells results in a functional defect that unleashes a potent anti-tumor response (25). It remains to be determined whether the differential requirements for p110δ and p110α in suppressing anti-tumor responses versus autoimmunity are due to different thresholds of PI3K activity required under these distinct pathophysiological processes or whether there are CNS- or inflammation-specific factors that favor a role for p110α in EAE and spontaneous neuropathy.

## Supplementary Material

Data Supplement
