# Supplementary material for: Loss of Phosphatidylinositol 3-Kinase Activity in Regulatory T Cells Leads to Neuronal Inflammation
Source: J Immunol. 2020 May 15;205(1):78–89. doi: 10.4049/jimmunol.2000043 (PMC7311201; doi:10.4049/jimmunol.2000043)
Supplement: Data Supplement [file JI_2000043.zip › JI_2000043_Supplemental_Figures_1.pdf]

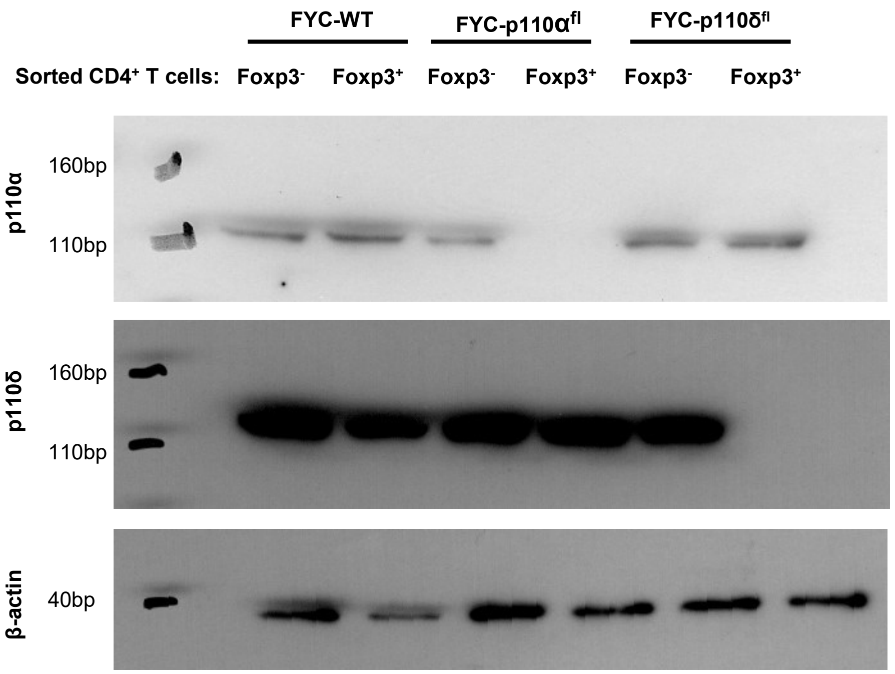


**Supplemental Figure 1: Treg cell-specific deletion of p110α and p110δ**

$CD4^+$ cells were immunomagnetically purified from pooled lymph node cells from FYC-WT, FYC-p110$\alpha^{fl}$, FYC-p110$\delta^{fl}$ and FYC-p110$\alpha^{fl}\delta^{fl}$ mice (8 mice per group). $YFP^-$ Tcon and $YFP^+$ Treg cell populations were flow-sorted and p110α and p110δ protein levels were detected in the cell homogenates by Western blot. (Images were cropped for concise presentation).

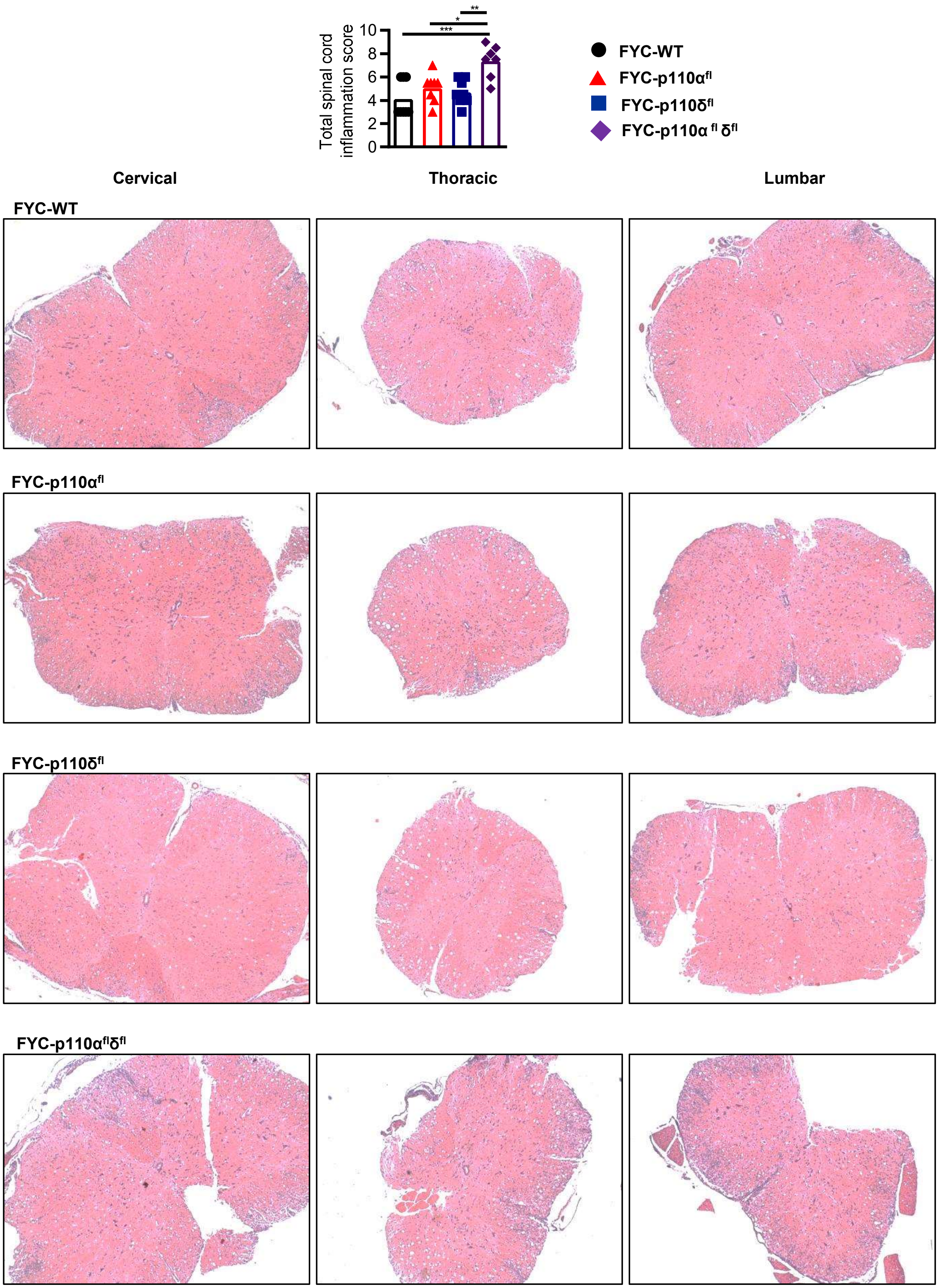


**Supplemental Figure 2: Simultaneous loss of p110α and p110δ in Treg cells results in increased spinal cord inflammation at peak EAE disease severity compared to Treg cell-specific loss of p110α or p110δ alone.**

Spinal cords were collected at 16-days post EAE induction. H&E stained sections were initially subjected to unblinded screening for lesions. Pathological findings, routine or otherwise, were then scored blindly on a semi-quantitative scale ranging from 0 (absent) to 5 (extensive/severe). (n=6; data representative of at least 2 independent experiments; Representative haematoxylin and eosin stained sections; all images are at x4 magnification).

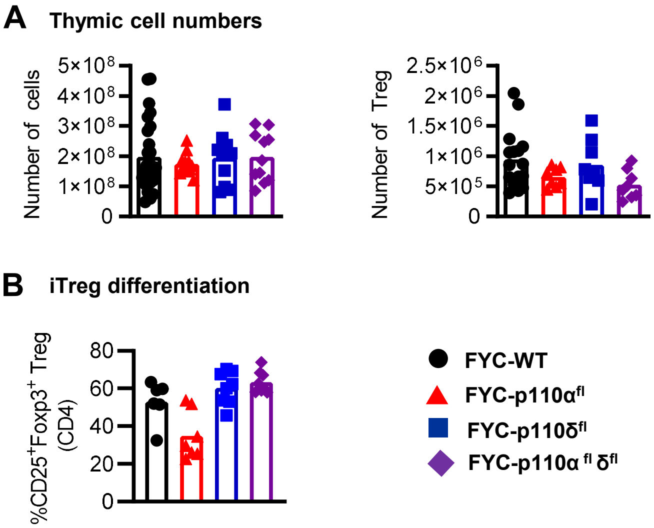


**Supplemental Figure 3: Deletion of p110α and/or p110δ in Treg cells does not alter Treg cell numbers in the thymus or *in vitro* iTreg differentiation**

**A**: Single cell suspensions were prepared from the thymi of naïve mice. Total thymus cell numbers were determined by automatic counting (CASY counter) and YFP$^+$ Treg cell numbers were determined by flow cytometry. (Combined results from 2 independent experiments, n=5-6 per experiment) **B**: Naïve CD4$^+$ T cells were cultured for 5 days in the presence of anti-CD3/anti-CD28 stimulation and Treg-polarising cytokines:10µg/mL anti-CD4 and anti-IFN-γ, 10ng/mL TGF-β, 20ng/mL IL-2. CD4$^+$ cells were identified by flow cytometry by excluding debris based on forward and side scatter, then gating on single cells, followed by live CD4$^+$ cells. (n=5-6; Results representative of at least two independent experiments).
